# Alzheimer’s disease: using gene/protein network machine learning for molecule discovery in olive oil

**DOI:** 10.1186/s40246-023-00503-6

**Published:** 2023-07-07

**Authors:** Luís Rita, Natalie R. Neumann, Ivan Laponogov, Guadalupe Gonzalez, Dennis Veselkov, Domenico Pratico, Reza Aalizadeh, Nikolaos S. Thomaidis, David C. Thompson, Vasilis Vasiliou, Kirill Veselkov

**Affiliations:** 1 Division of Cancer, Department of Surgery and Cancer, Faculty of Medicine, Imperial College London, London, UK; 2 Department of Emergency Medicine, Yale School of Medicine, New Haven, CT USA; 3 Department of Computing, Faculty of Engineering, Imperial College London, London, UK; 4 Prescient Design, Genentech | Roche, Basel, Switzerland; 5 Alzheimer’s Center at Temple, Lewis Katz School of Medicine, Temple University, Philadelphia, PA USA; 6 Department of Environmental Health Sciences, Yale University, New Haven, CT USA; 7 Laboratory of Analytical Chemistry, Department of Chemistry, National and Kapodistrian University of Athens, Panepistimiopolis Zografou, 15771 Athens, Greece

**Keywords:** Olive oil, Alzheimer’s disease, Network propagation, Nutrition

## Abstract

Alzheimer’s disease (AD) poses a profound human, social, and economic burden. Previous studies suggest that extra virgin olive oil (EVOO) may be helpful in preventing cognitive decline. Here, we present a network machine learning method for identifying bioactive phytochemicals in EVOO with the highest potential to impact the protein network linked to the development and progression of the AD. A balanced classification accuracy of 70.3 ± 2.6% was achieved in fivefold cross-validation settings for predicting late-stage experimental drugs targeting AD from other clinically approved drugs. The calibrated machine learning algorithm was then used to predict the likelihood of existing drugs and known EVOO phytochemicals to be similar in action to the drugs impacting AD protein networks. These analyses identified the following ten EVOO phytochemicals with the highest likelihood of being active against AD: quercetin, genistein, luteolin, palmitoleate, stearic acid, apigenin, epicatechin, kaempferol, squalene, and daidzein (in the order from the highest to the lowest likelihood). This in silico study presents a framework that brings together artificial intelligence, analytical chemistry, and omics studies to identify unique therapeutic agents. It provides new insights into how EVOO constituents may help treat or prevent AD and potentially provide a basis for consideration in future clinical studies.

**Supplementary Information:**

The online version contains supplementary material available at 10.1186/s40246-023-00503-6.

## Introduction

Alzheimer’s disease (AD) is a chronic neurodegenerative disorder that manifests clinically with a progressive decline in cognitive functions and memory. The disease is characterized primarily by the pathological presence of deposits of misfolded proteins (i.e., amyloid beta peptides and phosphorylated microtubule-associated tau protein) and cell loss in brain areas important for language and memory. Despite decades of research, the exact pathogenesis of AD remains unknown and there exist no treatments to reverse or prevent its neurocognitive decline [1]. For this reason, worldwide the disease exerts a profound impact on the affected individuals and the caregivers, society, and healthcare systems that support them. Given the profound human, social, and economic burden that AD causes, the limited understanding of its pathophysiology, and lack of effective treatment modalities, it behooves researchers to consider novel and unconventional therapies for prevention and treatment of this disease [2].

In recent years, there has been growing interest in the potential neuroprotective effects of extra virgin olive oil (EVOO). This stems from EVOO being a key component of the Mediterranean diet, the consumption of which has been associated with a lower incidence of dementia and cognitive decline in large epidemiological studies [3–5]. Moreover, several interventional clinical studies have also shown promising results in the use of olive oil and EVOO phytochemicals to improve cognitive outcomes in individuals with AD [6–8]. However, the specific phytochemicals responsible for these effects remain unknown due to the complex nature of EVOO [5, 7, 9], and the time and cost associated with isolating sufficient quantities of individual constituents and examining their effects on the development and progression of AD.

Different methods have been used to identify bioactive molecules in food that may have therapeutic potential, particularly for complex diseases like AD. One of the most prominent computational approaches involves investigation of bioactive food molecules based on their structural similarity to the approved or experimentally validated drugs for treatment. This approach assumes that similarities in the chemical structures of food molecules and drug molecules would result in similar biological effects [10]. However, this method has its limitations. Even minor changes in a molecule’s chemical structure can lead to significant differences in biological effects. Consequently, a subtly modified molecule may not possess the same therapeutic potential as the original compound, or it may even cause undesirable side effects. The other approach involves exploration of bioactive food molecules by examining their individual protein targets, an approach often referred to as “one disease–one target–one drug” [11]. This reductionist approach to therapeutic intervention development links a specific disease to a single target molecule (such as a protein or enzyme), which is then modulated by a single drug or food molecule to treat the disease. The underlying idea is that a disease can be understood as a consequence of a single molecular dysfunction, and that correcting this dysfunction can lead to the cure or management of the disease. In such a scenario, the drug or food molecule would interact specifically with the target molecule in a specific manner (e.g., inhibiting its activity, enhancing its activity, or modulating it in some other way) that intervenes with the disease process. This approach has proven to be successful in some cases, such as the development of targeted therapies for certain types of cancer. For example, pembrolizumab (Keytruda®) and nivolumab (Opdivo®) drugs target the programmed cell death protein 1 (PD-1) pathway, which helps cancer cells evade the immune system. They are used to treat various types of cancer, including melanoma, non-small cell lung cancer, and head and neck cancer [12]. Generally, “one disease–one target–one drug/food molecule” approach may be of more limited utility for complex and multifactorial diseases (i.e., involving multiple molecular pathways and genetic factors).

Recent advances in artificial intelligence (AI), coupled with the explosive growth of large-scale, multi-source data on food, drugs, and diseases, offer a unique opportunity to identify molecules within foods that may prevent and/or reverse disease by using more holistic and systems-based approaches [13]. Among these, network machine learning [14, 15] and graph neural networks [16] have shown promising results in predicting bioactive molecules within foods based on their ability to target disease network (i.e., dysregulated genes and protein pathways). An example of this approach is the virus–host interaction network responsible for COVID-19 [17].

In the present study, we hypothesize that a successful prevention or treatment strategy for AD should focus on the modulation of multiple biochemical networks involved in its pathogenesis including amyloid beta production and aggregation, tau hyperphosphorylation, and neuroinflammation [18]. Drawing on our prior research investigating bioactive compounds in food against cancer [16] and COVID-19 [17], we utilized network-based machine learning methods and human protein–protein interaction data to identify EVOO-based bioactive molecules that could target AD.

## Methodology

Our approach is based on the modeling of the interplay between disease-causing proteins and proteins disrupted by drugs or EVOO phytochemicals, while accounting for network interactions. In simple terms, human biological processes can be seen as a complex network of interacting genes and proteins, with humans having over 20 thousand protein-encoding genes and millions of protein–protein interactions. This complex network is known as an interactome [19].

**Fig. 1:**
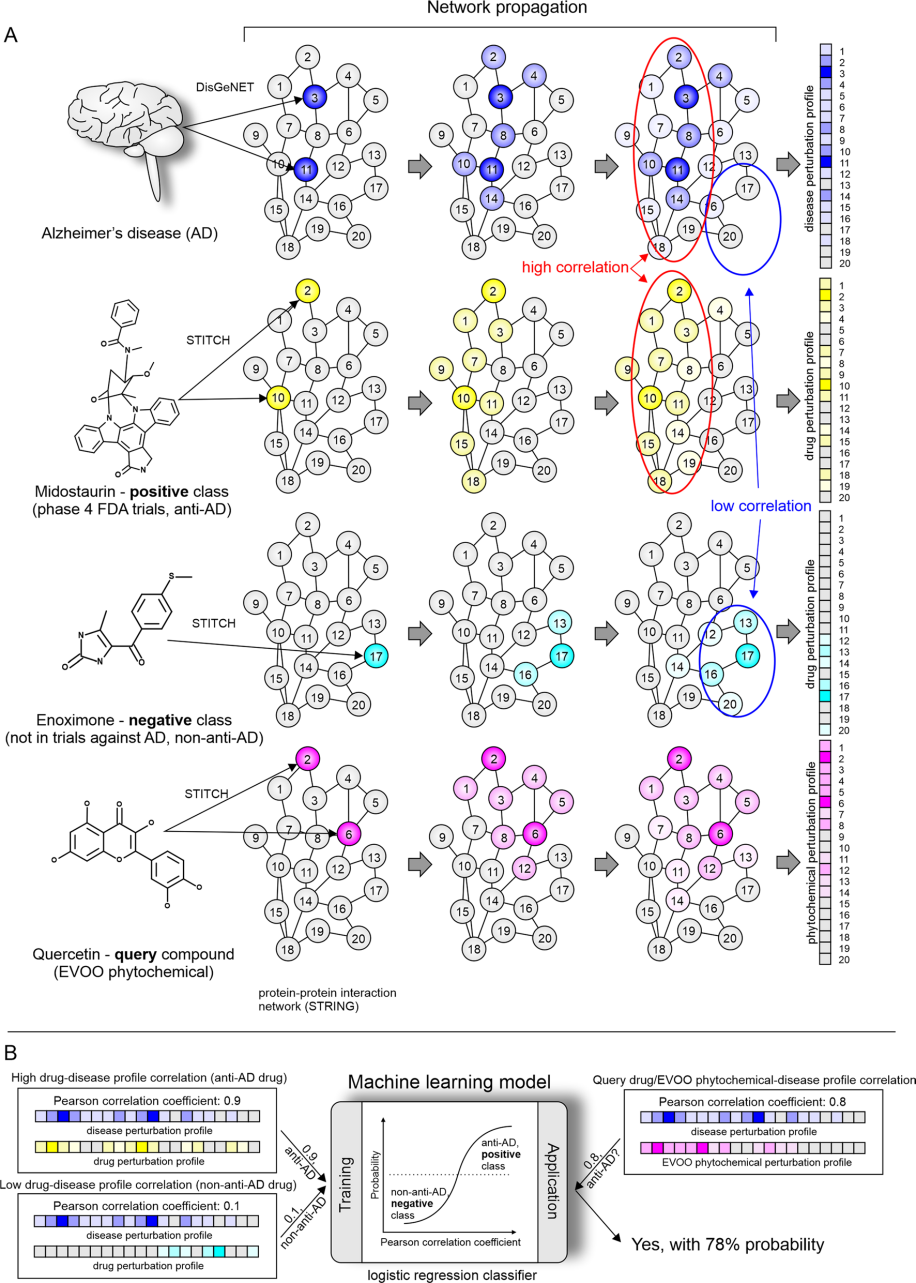
Summary of the methodology used to identify EVOO phytochemicals that potentially disrupt the interactome associated with AD onset and progression. Panel** A** illustrates the core principles of network propagation applied to generate protein perturbation profiles for disease, drugs, and EVOO phytochemicals. Human interactome is shown as a graph of interconnected nodes (numbered circles), where each node represents a protein and the edges (connections) represent protein–protein interactions. Numbers are arbitrarily chosen to serve as unique protein identifiers to allow for quick visual cross-referencing. Proteins directly affected by drugs or EVOO compounds were identified from the STITCH database and proteins directly dysregulated in AD were identified using DisGeNET database (as indicated by arrows on the left-hand side of panel **A**). The extent of a protein’s perturbation is reflected by the intensity of the color associated with the protein circle for AD (blue), an anti-AD drug in late-stage clinical trials (i.e., a positive class drug example like midostaurin) (yellow), a non-anti-AD drug (i.e., a negative class drug example like enoximone) (cyan), and the query EVOO compound (like quercetin) whose probability of being effective as anti-AD drug we are aiming to predict (magenta). Network propagation section illustrates how perturbation propagates through protein–protein connections in the network. The further the propagation spreads through the network, the less perturbation is generally expected unless many paths lead to the same hub proteins, amplifying the perturbation of these proteins. Color intensity indicates the level of perturbation. Highly correlated profile regions are shown in red ovals and the poorly correlated profile regions are shown in blue ovals. Panel **B** depicts an overall machine learning-based approach for predicting EVOO phytochemicals targeting the AD interactome in a similar way as do the advanced-stage anti-AD drugs in clinical trials. A logistic regression classifier was trained to discriminate between anti-AD drugs (positive class) and non-anti-AD drugs (negative class) based on profile correlations, which was then used to predict the probability of EVOO phytochemicals exhibiting anti-AD properties

In a healthy state, the system can be represented as a baseline or ground level. Disease-causing protein dysfunctions would disturb or disrupt their biochemical and signaling processes; this can be represented as a perturbation from the baseline. Such a disturbance would impact other proteins through protein–protein interactions, leading to a chain reaction, known as “network propagation” [14]. As a result, a single protein dysfunction can influence many other proteins and pathways in a cascade of perturbations. Different proteins will be impacted differently, depending on how “far” they are in the interaction network from the dysregulated protein. We have modeled the extent of these perturbations for over 20,000 proteins by using the known dysfunctional proteins linked to AD as starting perturbation points and employing a network propagation technique called “random walk with restarts” [20]. The resulting > 20,000 levels of protein disruption are referred to as a *disease perturbation profile* of the AD. Similarly, drugs or phytochemicals can interact with specific proteins and their effect on the ground state of those proteins can propagate through the network. In this case, the overall levels of perturbation of proteins are referred to as a *drug* or *phytochemical perturbation profile* (see Fig. 1A).

The methodology employed in the present study assumes that drugs and EVOO phytochemicals with anti-AD properties influence the proteins and pathways involved in the AD onset and progression. However, the disease and the drugs/phytochemicals do not have to target the exact same genes or protein, but rather share common network regions connected to them. To exert an anti-AD effect, a drug perturbation profile or phytochemical perturbation profile would be expected to be similar (but not necessarily identical) to the AD disease perturbation profile. A drug that does not affect AD (i.e., non-anti-AD) would be expected to have a perturbation profile that shares little overlap with the AD disease perturbation profile. To determine the level of similarity necessary to identify a unique drug/phytochemical candidate with anti-AD properties, the machine learning predictive model is trained using drugs expected to intervene with AD (anti-AD drugs, e.g., midostaurin) and drugs that do not affect AD (non-anti-AD drugs, e.g., enoximone) as positive class and negative class references, respectively (Fig. 1A, B and Sect. “Model training and application”).

### AD protein-encoding gene selection

In the present study, we identified proteins involved in AD using the extensive public dataset called DisGeNET [21]. From this dataset, 3,397 protein-encoding genes were found and then manually refined using expert-curated databases to ensure high-quality data. The databases used included UniProt [22], Comparative Toxicogenomics Database [23], Orphanet [24], Clinical Genome Resource [25], Genomics England PanelApp [26], Cancer Genome Interpreter [27], and the Psychiatric Disorders Gene Association Network [28].

Our study concentrated on proteins that influence AD onset or development, excluding those marked as therapeutic to exclude circularity in the analysis. This selection process led to the identification of 101 “key” protein-encoding genes strongly associated with AD risk and progression. These “key” genes were then mapped onto the STRING database [16] which provides a list of known protein–protein interactions for various organisms, along with their confidence scores (0–999). This step resulted in identification of 73 “key” AD proteins (Additional File 1). Protein–protein interactions were filtered later by thresholding the confidences scores, as part of the machine learning process (described below in the Methods Sect. “Network propagation”).

### EVOO phytochemical selection

We have developed a comprehensive list of 782 phytochemicals present in extra virgin olive products, including EVOO, table olives, and olive leaves (Additional File 2). This list includes plant-based chemicals and excludes metabolic intermediates, such as glucose [29]. Of these 782 phytochemicals, only those unique to EVOO or found in high concentrations were selected [30]. This selection was done by searching the literature for evidence of these chemicals in the olive oil matrix, a screening step that resulted in the identification of 117 EVOO “marker” phytochemicals.

DrugBank [31] and DrugCentral [32] databases were used to compile a list of experimental and available FDA-approved drugs, and FoodDB [33] database was used to compile a list of known phytochemicals (as described previously [16]). The STITCH database [34] was then used to find human proteins with which the molecules could interact, resulting in 67 EVOO marker phytochemicals. The database provides a confidence score (0–999) for each chemical–protein interaction, which is a measure of the likelihood of a true interaction based on the available evidence. In the present study, a fixed confidence cutoff of 200 was used, meaning that only interactions with a score of 200 or higher were considered. This threshold was chosen to ensure that the interactions being studied were more likely to be biologically relevant and supported by experimental evidence rather than only predicted computationally.

### Network propagation

After compiling the lists of drugs and EVOO phytochemicals and their impacted genes/proteins, and identifying the dysfunctional proteins associated with AD, we employed our previously developed network propagator tool (used in cancer [16] and COVID-19 [17] projects) to perform “random walk with restarts” [20]. This step enabled us to generate drug, EVOO phytochemical and disease perturbation profiles based on the STRING protein–protein network, consisting of 1,048,574 connections between 20,256 human proteins (matched between STITCH and STRING databases).

The random walk propagation was executed independently for drugs/EVOO phytochemicals and for the AD interactomes, using a range of restart probability values and several protein–protein connection thresholds (STRING protein connection thresholds of 400, 600, and 800; restart probability parameter c ranging from 0.0001 to 1.0). This range was selected based on our previous experience with random walk propagation in application to cancer and COVID-19 [16, 17]. Pearson correlation coefficients were calculated to assess the extent of overlap between the AD perturbation profile and each of the drug or EVOO phytochemical perturbation profiles. For each molecule–disease pair, a total of 2,160 Pearson correlation coefficients were calculated with different propagation settings, allowing for the assessment of molecule–disease perturbation similarities.

### Model training and application

Leveraging molecule–disease perturbation similarities, the machine learning models were trained to predict which FDA-approved drugs, of all those available in in DrugBank and DrugCentral (*N* = 1,802), are undergoing advanced stages (3 and 4) of FDA clinical trials (*N* = 32) for the treatment of AD. These 32 drugs are FDA-approved for other disease states and are being re-evaluated as potential therapeutic agents for the treatment of AD. A logistic regression classifier (based on the scikit-learn Python library for machine learning [35]) was fitted to the calculated Pearson correlation coefficients for each propagation settings from the previous step. The best settings were selected using fivefold stratified cross-validation. This approach is similar to the one we employed to predict phytochemicals with anticancer properties [16]. The overall predictive capacity of this approach was validated using 25 repeats of fivefold stratified cross-validation, with the optimal predictive model/propagation setting being selected by the internal fivefold stratified cross-validation.

A final set of models were identified that exhibited individual balanced accuracies higher than the cross-validation one; this resulted in 64 models. These models were then used to predict the probabilities of EVOO phytochemicals being able to exhibit anti-AD properties based on the correlation between their perturbation profiles and the perturbation profile of AD. Predicted probabilities were averaged between the selected models to provide a more robust ensemble-based probability prediction, further referred to as the correlation probability (%).

### Pathway analysis

The methodology was completed using GSEA Prerank [36] to analyze the top 10 approved drugs and EVOO (extra virgin olive oil) phytochemicals predicted by our model to have the highest correlation probability. We examined significant KEGG 2023 pathways [18] (*p* value < 0.001) for each molecule and ranked them based on their *p* values. KEGG (Kyoto Encyclopedia of Genes and Genomes) pathways are a collection of databases and tools that help researchers understand the biological functions, interactions, and networks within cells at a systems level. These pathways represent the molecular interactions and reaction networks for various cellular processes, such as metabolism, genetic information processing, environmental information processing, cellular processes, organismal systems, and human diseases. KEGG pathways help to visualize and interpret complex biological data, making it easier for researchers to understand how genes and proteins interact in different cellular contexts [18].

In this type of analysis, the pathway’s enrichment score measures the extent to which a particular biological process is overrepresented among the genes or proteins being studied. The nominal p value indicates the likelihood of observing the enrichment score by chance (assuming the gene/protein ranking is random and unrelated to the biological process under investigation) [18]. We calculated the interaction between AD proteins and EVOO phytochemical targets by multiplying the AD and EVOO phytochemical vectors. This product was then used in the GSEA analysis.

All data and Python scripts for the modeling, analysis, and figures are available from this Bitbucket repository: *bitbucket.org/iAnalytica/ai4olive*.

## Results

Our model allowed us to predict with 70.3% ± 2.6% accuracy which previously FDA-approved drugs were in phase 3 and 4 trials for AD specifically versus all other FDA-approved drugs not in AD trials. The resulting 64 models were used for scoring the EVOO phytochemicals; the probabilities of these phytochemicals predicted to be similar to the drugs in FDA phase 3 and 4 trials were then averaged to produce the final consensus prediction. EVOO phytochemicals with the highest probability of being like compounds in FDA trials were considered most likely to be biologically active.

Using our model, the FDA-approved drugs for other diseases (i.e., non-anti-AD drugs) were re-evaluated and ranked according to their correlation probability as having the highest probability of affecting AD (Additional File 3). Analysis of the most common pathways targeted by these drugs was *Alzheimer’s disease* (10), *Olfactory Transduction* (10), *Insulin Signaling* (5), *Phosphatidylinositol Signaling System* (2) and *Long-Term Potentiation* (1). The numbers in parentheses represent the number of times each pathway was identified for these drugs with a statistically significant p value for involvement with AD.

**Fig. 2:**
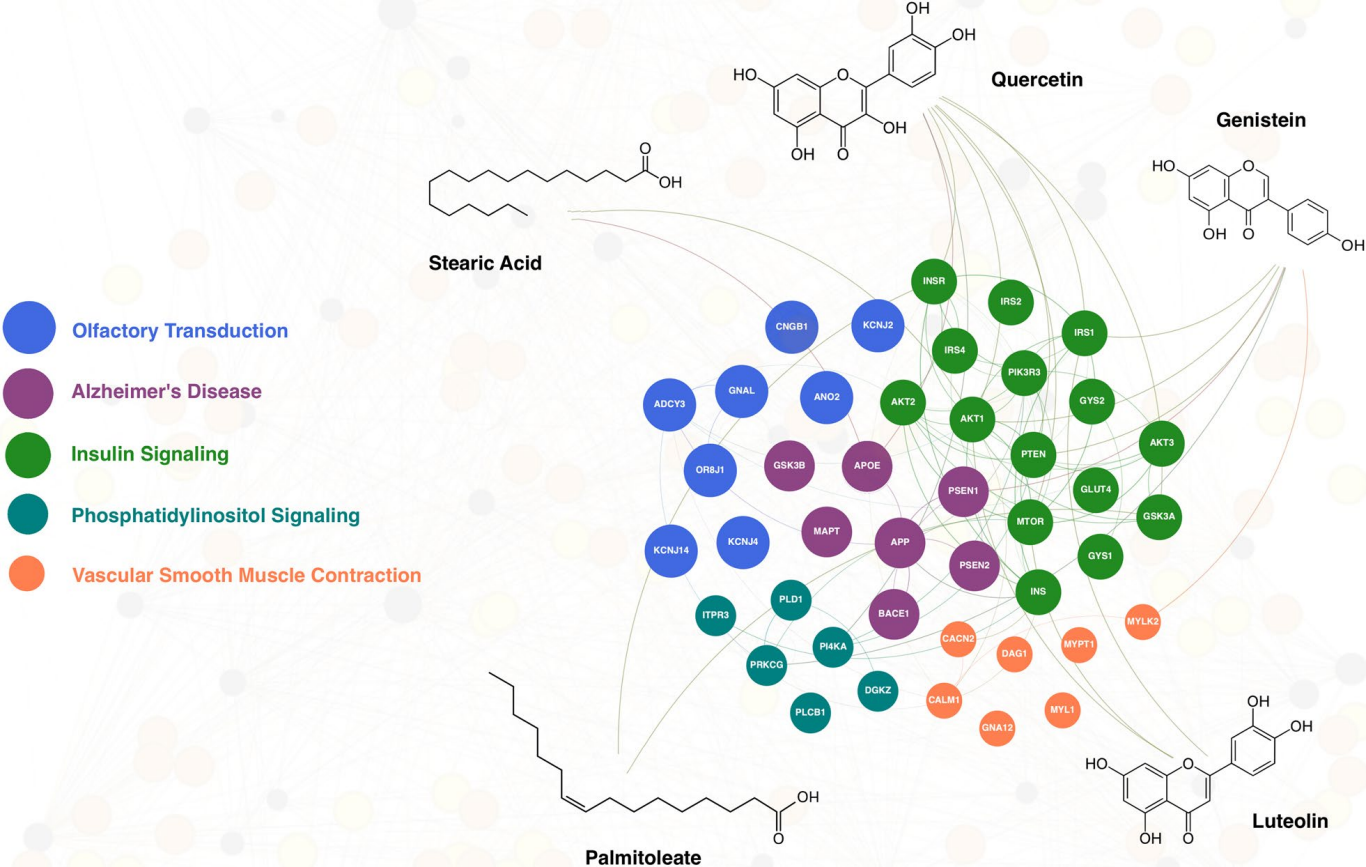
Predicted EVOO phytochemicals targeting dysregulated pathways in AD. The targeted pathways include: Blue denotes (Olfactory Transduction), violet denotes (Alzheimer’s disease), green denotes (Insulin Signaling), greenish blue denotes (Phosphatidylinositol Signaling), and orange denotes (Vascular Smooth Muscle Contraction). The key protein-encoding genes associated with a specific pathway (Additional File 5) are assigned the same color for easy identification. The size of each gene/protein node is proportional to how often the related pathway is targeted by the model EVOO phytochemicals listed in Table 1. Nodes associated with EVOO phytochemicals and their interactions with protein-encoding genes from AD dysregulated pathways are highlighted in brown. Only experimentally validated protein–protein interactions (STRING score greater than 800) are emphasized

Of the 67 EVOO marker phytochemicals (Additional File 4), quercetin, genistein, luteolin, palmitoleate, stearic acid, apigenin, epicatechin, kaempferol, squalene, and daidzein had the highest probability of interfering with AD (Table 1). The most common pathways predicted to be affected by these phytochemicals include *Olfactory Transduction* (10), *Alzheimer’s disease* (9), *Insulin Signaling Pathway* (7), *Phosphatidylinositol Signaling System* (5), and *Vascular Smooth Muscle Contraction* (3). As before, the numbers in parentheses represent the number of times each pathway was identified for these EVOO phytochemicals with a statistically significant p value for involvement with AD. Examples of the select EVOO phytochemicals and their connection with the most common AD pathways and involved proteins in our analysis are depicted in Fig. 2.

**Table 1:** EVOO marker phytochemicals predicted to have highest probability of influencing AD onset and/or progression

Rank ^a^	EVOO marker phytochemical	Correlation probability (%)^b^	Putative mechanism of action (Literature)^c^	Putative pathways affected^d^
1	Quercetin	78.0	Decreases oxidative stress, modulates cytokines, inhibits amyloid beta aggregation, and decreases tau phosphorylation [37–39]. (↑)	Alzheimer’s disease, Olfactory Transduction, Phosphatidylinositol Signaling System, Insulin Signaling Pathway
2	Genistein	75.5	Has antioxidant activity and ameliorates amyloid beta induced neurotoxicity induced by amyloid beta [40–42]. (↑)	Alzheimer’s disease, Olfactory Transduction, Insulin Signaling Pathway, Vascular Smooth Muscle Contraction
3	Luteolin	73.9	Modulates insulin resistance systemically and in the central nervous system and alters amyloid beta deposition [39]. (↑)	Olfactory Transduction
4	Palmitoleate	69.5	Preserves endothelial function, prevents endoplasmic reticulum stress, reduces lipogenesis and fatty acid desaturation in adipocytes, and suppresses cytokine production [43]. Decreases NF-KB nuclear translocation via the stimulation of PPARy and the phosphorylation of AMPK, which increases MGL2, IL-10, TGFß1, and MRC1 [40]. (≈)	Alzheimer’s disease, Olfactory Transduction, Insulin Signaling Pathway, Vascular Smooth Muscle Contraction
5	Stearic Acid	67.5	No consistent evidence	Alzheimer’s disease, Olfactory Transduction
6	Apigenin	67.4	Has anti-inflammatory and antioxidative activity, and downregulates BACE1 enzyme [44–46]. (↑)	Alzheimer’s disease, Olfactory Transduction, Long-Term Potentiation, Phosphatidylinositol Signaling System, Glioma, Insulin Signaling Pathway, GnRH Signaling Pathway, Neurotrophin Signaling Pathway
7	Epicatechin	66.4	Reduces amyloid beta concentrations in brain and serum and sequesters by-products of oxidation [47, 48]. (↑)	Alzheimer’s disease, Olfactory Transduction, Long-Term Potentiation, Phosphatidylinositol Signaling System, Insulin Signaling Pathway, Calcium Signaling Pathway
8	Kaempferol	65.1	Attenuates oxidative stress and inflammation through multiple mechanisms and delays memory loss [49, 50]. (↑)	Alzheimer’s disease, Olfactory Transduction, Phosphatidylinositol Signaling System, Insulin Signaling Pathway, Vascular Smooth Muscle Contraction
9	Squalene	63.7	No consistent evidence	Alzheimer’s disease, Olfactory Transduction, Maturity Onset Diabetes of the Young
10	Daidzein	62.1	Decreases amyloid beta aggregation and amyloid beta induced neuronal death and increase hippocampal neuronal cell proliferation [51, 52]. (↑)	Alzheimer’s disease, Olfactory Transduction, Glioma, Phosphatidylinositol Signaling System, Long-Term Potentiation, Insulin Signaling Pathway, mTOR Signaling Pathway
Mean		**68.9**		

^a^Phytochemicals were ranked from highest to lowest score based on correlation probability

^b^Logistic regression was used to calculate the correlation probability. It predicts the likelihood of an EVOO marker phytochemical having an effect on AD

^c^Arrow at the end of each identified mechanism of action indicates whether most evidence found was positive (↑), equivocal (≈), or negative (↓) toward preventing or halting the onset and/or progression of AD

^d^Statistically significant KEGG 2023 pathways (GSEA nominal *p* value < 0.001) ordered from the lowest to the highest *p* values

## Discussion

Using machine learning, the present study identified FDA-approved drugs and EVOO phytochemicals that are likely to interact with genes and proteins associated with AD pathophysiology. The model was initially trained to differentiate between drugs approved by the FDA in phases 3 or 4 to treat AD (= positive class) versus those drugs approved by the FDA to treat other diseases aside from AD (= negative class). This was the model used to predict which EVOO phytochemicals might affect the development and/or progression of AD.

The correlation probability scores obtained for model EVOO phytochemicals were similar to those obtained for the FDA-approved phase 3 or 4 drugs, suggesting that the phytochemicals interact with the same pathways as the FDA-approved drugs.

The use of machine learning to identify drug targets using existing databases is only in its infancy and the accuracy of the predictions rests in the quality of the databases being used (which are always being updated as new knowledge is generated). The results obtained in the present study using network propagation (random walk with restarts) support the validity of this approach in that several of the identified EVOO phytochemicals, such as quercetin, have been previously found to have a positive effect on preventing the development or progression of AD (see discussion below). For example, quercetin, the EVOO phytochemical shown to have the strongest correlation probability in the present study, has previously been evaluated as a neuroprotective agent in AD [38]. It is thought to be protective against AD by decreasing oxidative stress, modulation of cytokines, inhibiting amyloid beta aggregation, and decreasing tau phosphorylation [37–39]. Genistein, apigenin, catechin (also called epicatechin), kaempferol, similarly, have also been identified as potential therapeutic agents for AD [40–42, 44–50]. The flavonoids luteolin and daidzein, however, are novel agents which have not been investigated as a therapeutic in AD. Palmitoleate and stearic acid have undergone limited evaluation for their biologic actions in the central nervous system [53–56]. To date, squalene has not been considered a therapeutic phytochemical, although its metabolism has been considered in the pathogenesis of AD [57–59].

Eleven different KEGG pathways were identified as being targeted by the ten EVOO marker phytochemicals deemed most likely to interact with AD (Table 1). The pathways with the highest number of occurrences included *Olfactory Transduction*, *Alzheimer’s disease*, *Insulin Signaling Pathway*, *Phosphatidylinositol Signaling System*, and *Vascular Smooth Muscle Contraction*, respectively. The *Alzheimer’s disease* pathway includes the accumulation of beta-amyloid peptides, the formation of neurofibrillary tangles, oxidative stress, inflammation, and neuronal apoptosis events [18]. *Olfactory Transduction* is not known to be directly related to AD, although there is some evidence to suggest that changes in olfactory function may be an early marker of the disease. For example, AD-affected individuals often have a reduced sense of smell [60, 61], and olfactory dysfunction may precede the onset of cognitive symptoms by several years [62]. In addition, there is evidence to suggest that the accumulation of amyloid beta protein (a hallmark of AD) may disrupt olfactory function by interfering with the olfactory transduction pathway [63]. The *Insulin Signaling Pathway* is involved in several aspects of AD pathology, including amyloid beta and tau metabolism, neuroinflammation, and synaptic plasticity [64]. *Phosphatidylinositol Signaling System* pathway plays a crucial role in the regulation of various cellular processes, such as cell growth, proliferation, and survival [65]. In AD, this system has been implicated in the regulation of amyloid beta production, neuroinflammation, and synaptic dysfunction, and, as such, its dysregulation may contribute to the pathological processes that underlie AD [65]. Finally, *Vascular Smooth Muscle Contraction* (VSMC) is crucial for blood flow and pressure regulation. It is implicated in AD progression, as cerebrovascular dysfunction can lead to impaired blood flow, reduced brain oxygen supply, and increased AD symptoms and neuronal damage [66]. Amyloid beta accumulation can also disrupt blood vessels and impair VSMC, limiting their response to blood flow and pressure changes and escalating the risk of hypoperfusion and neurovascular damage [67].

The analytical approach taken in the present study has several limitations, many of which are due to the nature of the datasets underlying our data inputs. For example, the exact pathogenesis of AD is unknown. If there are proteins and genes that have a profound impact on AD development but are as yet undiscovered, they will not be accounted for in this study. The STITCH and STRING databases are themselves incomplete, and while it is unlikely, it is possible there are proteins, phytochemical–protein, or protein–protein interactions known to have an impact in AD which are not included in those databases. As these databases improve with advancing knowledge, that approach taken in the present study should become more powerful. Furthermore, we only analyzed a subset of AD expert-curated genes; it is possible that the 73 we selected do not fully characterize the disease process. Our analysis also does not discern whether the identified EVOO phytochemicals are necessarily effective against AD or whether their effects would be anti-AD or pro-AD, although they are more likely to be as effective as the phase 3 and 4 FDA-approved drugs currently undergoing AD clinical trials than any other molecules found in EVOO. It is also important to recognize that the number of these positive class drugs (i.e., FDA-approved drugs undergoing AD trials) was much smaller (32) than number of negative class drugs (i.e., FDA-approved non-anti-AD drugs) that were used for model training (1,745). Such an unbalanced dataset increases the likelihood of bias toward the negative class, leading to underestimation of EVOO phytochemicals that may influence AD. With identification of more anti-AD drugs and updated databases in the future, it should be possible to further improve the current model’s predictive capacity.

We hope our *in silico* work will inspire further studies to experimentally address the existing limitations, to validate our findings, and to fully evaluate the EVOO role in the prevention of AD.

## Conclusion

It is well known that diet and lifestyle influence health. Machine learning is a novel, cost-effective way to evaluate the potential health benefits of individual EVOO phytochemicals. The present study provides an approach that brings together artificial intelligence, analytical chemistry, and omics studies to explore the interactions of phytochemicals with pathways involved in a disease states, information that can lead to the identification of novel therapeutic entities in a natural product (that contains a heterogeneous mixture of phytochemicals). The analyses identified several individual EVOO phytochemicals that have a high likelihood of interfering with AD, a few of which (e.g., quercetin, genistein) have shown promising effects on AD pathogenesis. Others (e.g., luteolin) are worthy of further in vitro and in vivo study. It is only through the conduct of such studies will the predictive utility of our machine learning approach be validated. While the results of the present study shed light on how EVOO may help treat or prevent AD, the same approach may be applied to identify EVOO phytochemicals (or other food constituents) that treat other diseases, such as hypertension or dyslipidemia.

## Supplementary Information

**Additional file 1** List of 73 AD genes considered to be dysregulated in AD.

**Additional file 2** List of 782 phytochemicals present in extra virgin olive products, including EVOO, table olives, and olive leaves. It includes both EVOO markers and non-markers.

**Additional file 3** 10 FDA-approved drugs and not undergoing AD clinical trials that were classified the highest by our model.

**Additional file 4 ** 67 EVOO marker phytochemicals predicted and ranked according to the highest probability of influencing AD onset and/or progression.

**Additional file 5** Detailed examination of the pathways targeted by phytochemicals in EVOO, which are associated with AD and have been highly predicted by our network propagation model.
