# Supplementary material for: Alzheimer’s disease: using gene/protein network machine learning for molecule discovery in olive oil
Source: Hum Genomics. 2023 Jul 7;17:57. doi: 10.1186/s40246-023-00503-6 (PMC10327379; doi:10.1186/s40246-023-00503-6)
Supplement: Supplementary file 3 — Additional file 3 10 FDA-approved drugs and not undergoing AD clinical trials that were classified the highest by our model. [file 40246_2023_503_MOESM3_ESM.docx]

**Additional File 3 - Approved Drugs Repurposing**

**Table 1** Top FDA approved drugs and not undergoing AD clinical trials that were classified the highest by our model.

| **Rank ^a^** | **Approved Drug** | **Correlation Probability (%) ^b^** | **Putative Mechanism of Action (Literature) ^c^** | **Putative Pathways Affected ^d^** |  |
| --- | --- | --- | --- | --- | --- |
| 1 | Cyclosporine | 78.8 | Inhibits calcineurin, while reducing inflammation and oxidative stress (↑) [75] | **Alzheimer’s Disease**, Olfactory Transduction, Insulin Signaling Pathway | |
| 2 | Edetic Acid | 78.4 | Chelates metals such as aluminum, which may be involved in Alzheimer's pathology (↓) [76] | **Alzheimer’s Disease**, Olfactory Transduction | |
| 3 | Diethylstilbesterol | 78.0 | Binds to and activates estrogen receptors, which have neuroprotective effects (↑) [77] | **Alzheimer’s Disease**, Olfactory Transduction | |
| 4 | Nitric Oxide | 77.9 | Regulates cerebral blood flow and modulates neurotransmitter release, improving cognitive function (↑) [78] | **Alzheimer’s Disease**, Olfactory Transduction, Long Term Potentiation, Insulin Signaling Pathway, GnRH Signaling Pathway, Vascular Smooth Muscle Contraction, Neurotrophin Signaling Pathway | |
| 5 | Streptomycin | 77.6 | Inhibits protein synthesis by binding to the bacterial ribosome, potentially reducing the risk of bacterial infections that may exacerbate Alzheimer's pathology (↑) [79] | **Alzheimer’s Disease**, Olfactory Transduction, Phosphatidylinositol Signaling System, Insulin Signaling Pathway | |
| 6 | Doxorubicin | 77.5 | Inhibits topoisomerase II and generates free radicals, leading to DNA damage and cell death. May also have neuroprotective effects (↓) [80] | **Alzheimer’s Disease**, Olfactory Transduction, Insulin Signaling Pathway | |
| 7 | Phosphoric acid | 77.5 | Modifies proteins, potentially reducing the formation of beta-amyloid plaques in the brain (↑) [81] | **Alzheimer’s Disease**, Olfactory Transduction | |
| 8 | Valproic Acid | 77.5 | Inhibits histone deacetylases, which can lead to the upregulation of genes involved in synaptic plasticity and memory formation (↑) [82] | **Alzheimer’s Disease**, Olfactory Transduction | |
| 9 | Ammonium chloride | 77.3 | Acidifies lysosomes, potentially reducing the accumulation of beta-amyloid and tau proteins in neurons (↑) [83] | **Alzheimer’s Disease**, Olfactory Transduction | |
| 10 | Potassium Chloride | 76.8 | Acts as an electrolyte in the brain, potentially modulating the activity of neurons and improving cognitive function (↑) [84] | **Alzheimer’s Disease**, Olfactory Transduction, Phosphatidylinositol Signaling System, Insulin Signaling Pathway | |
| **Mean** |  | **77.7** |  |  | |

^a^ Drugs were ranked from highest to lowest score based on correlation probability.

^b^ Logistic regression was used to calculate the correlation probability. It predicts the likelihood of a drug having an effect on AD.

^c^ Arrow at the end of each identified mechanism of action indicates whether most evidence found was positive (↑), equivocal (≈) or negative (↓) towards preventing or halting the onset and/or progression of AD.

^d^ Only statistically significant KEGG 2023 pathways (GSEA nominal p-value < 0.001) were represented for each drug and ordered from the lowest to the highest p-values.
