# Supplementary material for: Alzheimer’s disease: using gene/protein network machine learning for molecule discovery in olive oil
Source: Hum Genomics. 2023 Jul 7;17:57. doi: 10.1186/s40246-023-00503-6 (PMC10327379; doi:10.1186/s40246-023-00503-6)
Supplement: Supplementary file 5 — Additional file 5 Detailed examination of the pathways targeted by phytochemicals in EVOO, which are associated with AD and have been highly predicted by our network propagation model. [file 40246_2023_503_MOESM5_ESM.docx]

**Additional File 5 - Exploring the Effects of EVOO Molecules on Cellular Pathways through Interactome Analysis and Network Propagation**

The following section provides a detailed examination of the pathways targeted by phytochemicals in EVOO, which are associated with Alzheimer's disease (AD) and have been highly predicted by our network propagation model.

**1. Alzheimer’s Disease Pathway**

The main hallmarks of AD (as illustrated in the KEGG pathway) include amyloid-beta production and aggregation, tau hyperphosphorylation, and neuroinflammation:

Amyloid-Beta Production and Aggregation

AD is characterized by the accumulation of amyloid-beta (Aβ) peptides, which form extracellular plaques. Aβ peptides are generated from the amyloid precursor protein (APP) by sequential cleavage via beta-secretase (BACE1) and gamma-secretase complex (composed of presenilin, nicastrin, APH1, and PEN2). Mutations in APP or presenilin genes can lead to increased Aβ production, contributing to AD [1,2].

Tau Hyperphosphorylation

Intracellular neurofibrillary tangles, composed of hyperphosphorylated tau protein, are another hallmark of AD. Tau is a microtubule-associated protein, and its hyperphosphorylation destabilizes microtubules, leading to impaired axonal transport and neuronal dysfunction. Dysregulation of kinases, such as glycogen synthase kinase-3β (GSK-3β) and cyclin-dependent kinase 5 (CDK5), and phosphatases contributes to tau hyperphosphorylation in AD [3,4].

Neuroinflammation

In AD, activation of microglia and astrocytes leads to chronic neuroinflammation. Microglia, the resident immune cells of the brain, can become overactivated and release pro-inflammatory cytokines, such as interleukin-1β (IL-1β), tumor necrosis factor-alpha (TNF-α), and interleukin-6 (IL-6), which contribute to neuronal dysfunction and death [5,6].

**Table 1** Key proteins and complexes involved in the AD pathway.

| **Protein/Gene** | **Symbol** | **Function** |
| --- | --- | --- |
| Amyloid precursor protein | APP | Cleavage of APP by β-secretase and γ-secretase produces amyloid-β (Aβ) peptides, which aggregate to form amyloid plaques, a hallmark of AD. |
| Presenilin 1 | PSEN1 | Catalytic component of the γ-secretase complex, responsible for the cleavage of APP to generate Aβ peptides. |
| Presenilin 2 | PSEN2 | Catalytic component of the γ-secretase complex, responsible for the cleavage of APP to generate Aβ peptides. |
| Beta-site APP cleaving enzyme 1 | BACE1 | Also known as β-secretase, BACE1 is an enzyme involved in the cleavage of APP, producing Aβ peptides. |
| Apolipoprotein E | APOE | APOE is involved in lipid metabolism and is associated with an increased risk of developing AD, especially the APOE ε4 allele. |
| Tau protein | MAPT | Hyperphosphorylated tau protein forms neurofibrillary tangles, another hallmark of AD, leading to neuronal dysfunction and cell death. |
| Glycogen synthase kinase 3 beta | GSK3B | Involved in tau protein hyperphosphorylation, contributing to the formation of neurofibrillary tangles. |

**2. Olfactory Transduction**

The relationship between olfactory transduction and AD is not direct, but there is evidence to suggest that olfactory dysfunction may be an early symptom of Alzheimer's and other neurodegenerative diseases. The connection between the olfactory system and AD may be related to the presence of pathological hallmarks in the olfactory system, as well as the proximity of the olfactory bulb to brain regions affected by AD.

Olfactory Dysfunction

Studies have shown that individuals with AD often experience a decline in their ability to smell. Olfactory dysfunction can precede cognitive impairment and memory loss and may serve as an early warning sign for AD [7,8].

Presence of AD pathology in Olfactory Regions

The olfactory bulb and olfactory cortex are among the first regions in the brain to be affected by Alzheimer's pathology, such as amyloid-beta plaques and neurofibrillary tangles. This may contribute to the observed olfactory dysfunction in Alzheimer's patients [9].

Proximity to Brain Regions Affected by AD

The olfactory bulb is anatomically close to the entorhinal cortex and hippocampus, which are critical for memory formation and are among the first regions to be affected in AD. This proximity might contribute to the spread of Alzheimer's pathology [10].

In summary, while the olfactory transduction pathway is not directly involved in AD, olfactory dysfunction may serve as an early symptom and is associated with the presence of Alzheimer's pathology in olfactory regions. Further research is needed to better understand the relationship between olfactory dysfunction and AD, and to determine if interventions targeting the olfactory system could help prevent or slow the progression of AD.

**Table 2** Key proteins and complexes involved in the Olfactory Transduction pathway.

| **Protein/Gene** | **Symbol** | **Function** |
| --- | --- | --- |
| Odorant molecules | - | Bind to olfactory receptors on the surface of olfactory receptor neurons in the nose. |
| Olfactory receptors | ORs (OR8J1) | G-protein coupled receptors (GPCRs) that bind odorant molecules and activate the downstream signaling cascade. |
| G-protein (Golf) | GNAL | Activates adenylate cyclase upon binding to an activated olfactory receptor. |
| Adenylate cyclase 3 | ADCY3 | Converts ATP to cyclic AMP (cAMP) in response to G-protein activation. |
| Cyclic AMP (cAMP) | - | Second messenger molecule that activates cyclic nucleotide-gated ion channels. |
| Cyclic nucleotide-gated ion channels | CNGA2, CNGA4, CNGB1 | Non-selective cation channels that open in response to cAMP, allowing the influx of Na+ and Ca2+ ions, leading to membrane depolarization. |
| Ca2+-activated Cl- channels | ANO2 | Activated by the influx of Ca2+ ions, mediating Cl- efflux to amplify the receptor potential. |
| Inward rectifier K+ channels | KCNJ2, KCNJ4, KCNJ14 | Help to repolarize the membrane potential, terminating the olfactory response. |

**3. Insulin Signaling Pathway**

The connection between the insulin signaling pathway and AD (AD) has been increasingly studied in recent years. AD has even been referred to as "type 3 diabetes" due to the involvement of insulin resistance and glucose metabolism dysfunction in the brain. The main connections between the insulin signaling pathway and AD are as follows:

Insulin resistance

Insulin resistance in the brain impairs insulin signaling, leading to reduced glucose uptake and energy metabolism. This can contribute to neuronal dysfunction and synaptic loss, which are characteristic of AD [11].

Amyloid-beta (Aβ) Metabolism

Impaired insulin signaling may affect the clearance and accumulation of Aβ peptides, which are the main components of amyloid plaques in AD. Insulin has been shown to regulate the expression and activity of enzymes responsible for Aβ production and degradation [12].

Tau Hyperphosphorylation

Insulin signaling can modulate tau phosphorylation through the activation of protein kinase B (Akt). Impaired insulin signaling may lead to the dysregulation of Akt and other kinases, promoting tau hyperphosphorylation and the formation of neurofibrillary tangles, another hallmark of AD [13].

Inflammation

Impaired insulin signaling can contribute to chronic inflammation in the brain, which has been implicated in the progression of AD. Inflammation can exacerbate Aβ accumulation and tau hyperphosphorylation, leading to further neuronal dysfunction and cell death [14].

Neuronal Survival and Synaptic Plasticity

Insulin signaling plays a crucial role in maintaining neuronal survival and promoting synaptic plasticity. Impaired insulin signaling in the brain can lead to reduced neurotrophic support, increased oxidative stress, and impaired synaptic function, all of which contribute to the cognitive decline observed in AD [15].

In summary, the connection between the insulin signaling pathway and AD is multifaceted, involving insulin resistance, Aβ metabolism, tau phosphorylation, inflammation, and neuronal survival.

**Table 3** Key proteins and complexes involved in the Insulin Signaling pathway.

| **Protein/Gene** | **Symbol** | **Function** |
| --- | --- | --- |
| Insulin | INS | Hormone that binds to the insulin receptor, initiating the insulin signaling cascade. |
| Insulin receptor | INSR | Receptor tyrosine kinase that is activated upon insulin binding, autophosphorylates, and triggers downstream signaling. |
| Insulin receptor substrate 1-4 | IRS1, IRS2, IRS3, IRS4 | Adaptor proteins that, when phosphorylated by the insulin receptor, serve as docking sites for downstream signaling molecules. |
| Phosphoinositide 3-kinase | PIK3R3 | A lipid kinase that generates phosphatidylinositol (3,4,5)-trisphosphate (PIP3) upon activation by IRS proteins. |
| Phosphatase and tensin homolog | PTEN | A lipid phosphatase that negatively regulates the insulin signaling pathway by dephosphorylating PIP3. |
| Protein kinase B (Akt) | AKT1, AKT2, AKT3 | Serine/threonine kinase activated by PIP3, responsible for multiple downstream effects, including glucose uptake and glycogen synthesis. |
| Glycogen synthase kinase-3 | GSK3A, GSK3B | A kinase that phosphorylates and inhibits glycogen synthase; Akt inactivates GSK-3, allowing glycogen synthesis. |
| Mammalian target of rapamycin | mTOR | A serine/threonine kinase that regulates protein synthesis, cell growth, and metabolism; activated by Akt. |
| Glucose transporter type 4 | GLUT4 | A glucose transporter that is translocated to the cell membrane in response to insulin signaling, allowing glucose uptake. |
| Glycogen synthase | GYS1, GYS2 | Enzyme responsible for glycogen synthesis; its activity is regulated by the insulin signaling pathway. |

**4. Phosphatidylinositol Signaling System**

The phosphatidylinositol signaling system has been implicated in AD through several mechanisms. Although the connection is not yet fully understood, research suggests that dysfunction in the phosphatidylinositol signaling pathway may contribute to the development and progression of AD. The main connections between the phosphatidylinositol signaling system and AD are as follows [16]:

Aβ Production and Clearance

Phosphatidylinositol signaling, particularly PI3K/Akt/mTOR pathway, has been implicated in regulating amyloid precursor protein (APP) processing and Aβ production. Dysregulation of this pathway can affect APP metabolism, leading to increased Aβ production and accumulation of amyloid plaques in the brain [17].

Tau Phosphorylation

The PI3K/Akt/GSK-3β signaling axis is involved in the regulation of tau phosphorylation. Impairment of the phosphatidylinositol signaling pathway can lead to dysregulation of Akt and GSK-3β, promoting tau hyperphosphorylation and the formation of neurofibrillary tangles, another pathological hallmark of AD [18].

Synaptic Function and Plasticity

Phosphatidylinositol signaling plays a critical role in the regulation of synaptic function and plasticity. Alterations in phosphatidylinositol signaling can impair synaptic transmission, leading to synaptic loss and cognitive decline in AD [19].

Neuroinflammation

Dysregulation of the phosphatidylinositol signaling pathway can contribute to neuroinflammation, a process that has been implicated in the progression of AD. Inflammatory responses can exacerbate Aβ accumulation and tau hyperphosphorylation, leading to further neuronal dysfunction and cell death [20].

Neuronal survival and apoptosis

Phosphatidylinositol signaling, particularly the PI3K/Akt pathway, is crucial for neuronal survival and protection against apoptosis. Dysfunction in this pathway can lead to increased neuronal vulnerability and cell death, contributing to neurodegeneration in AD [21,22].

In summary, the connection between the phosphatidylinositol signaling system and AD involves multiple mechanisms, including Aβ production and clearance, tau phosphorylation, synaptic function, neuroinflammation, and neuronal survival. A better understanding of the relationship between the phosphatidylinositol signaling pathway and AD may provide new therapeutic targets for the prevention and treatment of AD.

**Table 4** Key proteins and complexes involved in the Phosphatidylinositol Signaling System pathway.

| **Protein/Gene** | **Symbol** | **Function** |
| --- | --- | --- |
| Phosphatidylinositol kinase | PI4KA | Enzymes responsible for the phosphorylation of phosphatidylinositol (PI) to generate PI(4)P and PI(4,5)P2. |
| Phosphoinositide 3-kinase | PIK3R3 | A lipid kinase that generates phosphatidylinositol (3,4,5)-trisphosphate (PIP3) by phosphorylating PI(4,5)P2. |
| Phospholipase C | PLCB1 | Enzyme that hydrolyzes PI(4,5)P2 to generate inositol 1,4,5-trisphosphate (IP3) and diacylglycerol (DAG), initiating downstream signaling. |
| Inositol 1,4,5-trisphosphate receptor | ITPR3 | Calcium channel that releases calcium from the endoplasmic reticulum upon binding IP3, initiating calcium signaling. |
| Diacylglycerol kinase | DGKZ | Enzyme that phosphorylates DAG to generate phosphatidic acid (PA), modulating DAG signaling. |
| Protein kinase C | PRKCG | A family of serine/threonine kinases activated by DAG and calcium, involved in the regulation of numerous cellular processes. |
| Phosphatase and tensin homolog | PTEN | A lipid phosphatase that negatively regulates the phosphatidylinositol signaling system by dephosphorylating PIP3. |
| Protein kinase B (Akt) | AKT1, AKT2, AKT3 | Serine/threonine kinase activated by PIP3, involved in cell survival, growth, and metabolism. |
| Phospholipase D | PLD1 | Enzyme that hydrolyzes PI to generate PA and inositol, involved in various cellular processes, such as vesicle trafficking and signal transduction. |

**5. Vascular Smooth Muscle Contraction Pathway**

While the vascular smooth muscle contraction pathway is not directly involved in the classic AD pathways, it has been associated with AD through indirect connections. The main connections between the vascular smooth muscle contraction pathway and AD are as follows [23]:

Cerebral Blood Flow and Vascular Dysfunction

Vascular smooth muscle cells play a critical role in regulating cerebral blood flow by controlling the diameter of blood vessels. In AD, vascular dysfunction and impaired cerebral blood flow have been observed, which can contribute to neuronal dysfunction, reduced glucose and oxygen supply, and cognitive decline. The dysfunction of proteins involved in vascular smooth muscle contraction can contribute to this impaired blood flow regulation in AD [24].

Blood-Brain Barrier (BBB) Integrity

Vascular smooth muscle cells are an essential component of the neurovascular unit, which plays a crucial role in maintaining the integrity of the blood-brain barrier. BBB dysfunction has been observed in AD, which may lead to increased infiltration of peripheral immune cells and neurotoxic substances into the brain, exacerbating inflammation and neuronal damage. Abnormal vascular smooth muscle contraction may contribute to this BBB dysfunction in AD [25].

Cerebral amyloid angiopathy (CAA)

CAA is a condition characterized by the accumulation of amyloid-beta (Aβ) in the walls of cerebral blood vessels. This accumulation can lead to vessel wall thickening, reduced vessel elasticity, and impaired vascular smooth muscle function, contributing to cerebral blood flow dysregulation in AD. Aβ deposition in the vascular smooth muscle cells can disrupt the vascular smooth muscle contraction pathway and contribute to vascular dysfunction in AD [26].

Hypertension and Cardiovascular Risk Factors

The dysfunction of the vascular smooth muscle contraction pathway can contribute to hypertension, which is a known risk factor for AD. Long-term hypertension can lead to vascular damage, inflammation, and impaired cerebral blood flow regulation, all of which may contribute to the development or progression of AD [27].

In summary, the connection between the vascular smooth muscle contraction pathway and AD is mostly indirect, involving cerebral blood flow regulation, blood-brain barrier integrity, cerebral amyloid angiopathy, and cardiovascular risk factors such as hypertension. A better understanding of these relationships may help identify novel therapeutic targets for preventing or treating AD.

**Table 5** Key proteins and complexes involved in the Vascular Smooth Muscle Contraction pathway.

| **Protein/Gene** | **Symbol** | **Role in AD Pathway** |
| --- | --- | --- |
| G protein-coupled receptors | GNA12 | Receptors that bind various ligands, initiating intracellular signaling cascades that modulate smooth muscle contraction. |
| Heterotrimeric G proteins | GNA12 | Transduce signals from GPCRs to downstream effectors, such as phospholipase C, adenylate cyclase, and ion channels. |
| Phospholipase C | PLCB1 | Enzyme that hydrolyzes phosphatidylinositol 4,5-bisphosphate (PIP2) to generate inositol 1,4,5-trisphosphate (IP3) and diacylglycerol (DAG). |
| Inositol 1,4,5-trisphosphate receptor | ITPR3 | Calcium channel that releases calcium from the sarcoplasmic reticulum upon binding IP3, increasing intracellular calcium levels. |
| Diacylglycerol | DAG1 | Activates protein kinase C (PKC), which phosphorylates various proteins involved in smooth muscle contraction. |
| Protein kinase C | PRKCG | Serine/threonine kinase that is activated by DAG and calcium, regulating proteins involved in smooth muscle contraction. |
| Voltage-gated calcium channels | CACNA1C | Channels that allow calcium influx in response to membrane depolarization, contributing to smooth muscle contraction. |
| Calcium/calmodulin complex | CALM1 | Calcium-binding protein that, upon calcium binding, activates myosin light chain kinase (MLCK) and other target proteins. |
| Myosin light chain kinase | MYLK2 | Enzyme that phosphorylates myosin light chain (MLC) upon activation by calcium/calmodulin, promoting smooth muscle contraction. |
| Myosin light chain phosphatase | PPP1R12A | Enzyme that dephosphorylates MLC, promoting smooth muscle relaxation. |
| Myosin light chain | MYL1 | Regulatory protein of myosin that, when phosphorylated, initiates the cross-bridge cycle and muscle contraction. |

**References**

1. Hardy J, Selkoe DJ. The Amyloid Hypothesis of AD: Progress and Problems on the Road to Therapeutics. Science (1979). 2002;297:353–6.

2. Selkoe DJ. AD: Genes, Proteins, and Therapy. Physiol Rev. 2001;81:741–66.

3. Lee VM-Y, Goedert M, Trojanowski JQ. Neurodegenerative Tauopathies. Annu Rev Neurosci. 2001;24:1121–59.

4. Iqbal K, Liu F, Gong C-X, Grundke-Iqbal I. Tau in Alzheimer Disease and Related Tauopathies. Curr Alzheimer Res. 2010;7:656–64.

5. Heneka MT, Carson MJ, Khoury J El, Landreth GE, Brosseron F, Feinstein DL, et al. Neuroinflammation in AD. Lancet Neurol. 2015;14:388–405.

6. Wyss-Coray T, Rogers J. Inflammation in Alzheimer Disease--A Brief Review of the Basic Science and Clinical Literature. Cold Spring Harb Perspect Med. 2012;2:a006346–a006346.

7. Murphy C. Olfactory and other sensory impairments in Alzheimer disease. Nat Rev Neurol. 2019;15:11–24.

8. Wilson RS, Arnold SE, Schneider JA, Tang Y, Bennett DA. The relationship between cerebral AD pathology and odour identification in old age. J Neurol Neurosurg Psychiatry. 2007;78:30–5.

9. Attems J, Walker L, Jellinger KA. Olfactory bulb involvement in neurodegenerative diseases. Acta Neuropathol. 2014;127:459–75.

10. Braak H, Braak E. Neuropathological stageing of Alzheimer-related changes. Acta Neuropathol. 1991;82:239–59.

11. de la Monte SM, Wands JR. AD is Type 3 Diabetes—Evidence Reviewed. J Diabetes Sci Technol. 2008;2:1101–13.

12. Zhao W-Q, Alkon DL. Role of insulin and insulin receptor in learning and memory. Mol Cell Endocrinol. 2001;177:125–34.

13. Lesort M, Jope RS, Johnson GVW. Insulin Transiently Increases Tau Phosphorylation. J Neurochem. 1999;72:576–84.

14. Craft S. Insulin Resistance and Alzheimers Disease Pathogenesis: Potential Mechanisms and Implications for Treatment. Curr Alzheimer Res. 2007;4:147–52.

15. Zhao W, Chen H, Xu H, Moore E, Meiri N, Quon MJ, et al. Brain Insulin Receptors and Spatial Memory. Journal of Biological Chemistry. 1999;274:34893–902.

16. Ando K, Erneux C, Homa M, Houben S, de Fisenne M-A, Brion J-P, et al. Dysregulation of Phosphoinositide 5-Phosphatases and Phosphoinositides in AD. Front Neurosci. 2021;15.

17. Razani E, Pourbagheri-Sigaroodi A, Safaroghli-Azar A, Zoghi A, Shanaki-Bavarsad M, Bashash D. The PI3K/Akt signaling axis in AD: a valuable target to stimulate or suppress? Cell Stress Chaperones. 2021;26:871–87.

18. Kitagishi Y, Nakanishi A, Ogura Y, Matsuda S. Dietary regulation of PI3K/AKT/GSK-3β pathway in AD. Alzheimers Res Ther. 2014;6:35.

19. Frere SG, Chang-Ileto B, Di Paolo G. Role of Phosphoinositides at the Neuronal Synapse. 2012. p. 131–75.

20. Kinney JW, Bemiller SM, Murtishaw AS, Leisgang AM, Salazar AM, Lamb BT. Inflammation as a central mechanism in AD. Alzheimer’s & Dementia: Translational Research & Clinical Interventions. 2018;4:575–90.

21. Long H-Z, Cheng Y, Zhou Z-W, Luo H-Y, Wen D-D, Gao L-C. PI3K/AKT Signal Pathway: A Target of Natural Products in the Prevention and Treatment of AD and Parkinson’s Disease. Front Pharmacol. 2021;12.

22. Morrison RS, Kinoshita Y, Johnson MD, Ghatan S, Ho JT, Garden G. Neuronal Survival and Cell Death Signaling Pathways. 2003. p. 41–86.

23. Aguilar-Pineda JA, Vera-Lopez KJ, Shrivastava P, Chávez-Fumagalli MA, Nieto-Montesinos R, Alvarez-Fernandez KL, et al. Vascular smooth muscle cell dysfunction contribute to neuroinflammation and Tau hyperphosphorylation in Alzheimer disease. iScience. 2021;24:102993.

24. Kisler K, Nelson AR, Montagne A, Zlokovic B V. Cerebral blood flow regulation and neurovascular dysfunction in Alzheimer disease. Nat Rev Neurosci. 2017;18:419–34.

25. Sharma C, Woo H, Kim SR. Addressing Blood–Brain Barrier Impairment in AD. Biomedicines. 2022;10:742.

26. Weller RO, Massey A, Newman TA, Hutchings M, Kuo Y-M, Roher AE. Cerebral Amyloid Angiopathy. Am J Pathol. 1998;153:725–33.

27. Iadecola C, Davisson RL. Hypertension and Cerebrovascular Dysfunction. Cell Metab. 2008;7:476–84.
